# HPV Vaccine Hesitancy and Uptake: A Conceptual Analysis Using Rodgers' Evolutionary Approach

**DOI:** 10.1111/jan.16653

**Published:** 2024-12-09

**Authors:** Grace K. Kyei, Evans F. Kyei, Rockson Ansong

**Affiliations:** ^1^ College of Nursing and Health Sciences University of Massachusetts Boston Boston Massachusetts USA; ^2^ Center for Substance Use Research and Related Conditions, Capstone College of Nursing The University of Alabama Tuscaloosa Alabama USA

## Abstract

**Aim:**

This study examines the factors contributing to HPV vaccine hesitancy using Rodgers's evolutionary concept analysis, focusing on the impact of uncertainties about the vaccine on HPV‐related diseases despite its availability.

**Design:**

Rodgers's evolutionary concept analysis was applied to explore factors leading to HPV vaccine hesitancy. The analysis included studies published in English over the past 5 years. Exclusions were studies older than 5 years, non‐English publications, and those focusing on other vaccines.

**Data Sources:**

A comprehensive literature review was conducted using key databases such as PsycINFO, CINAHL, MEDLINE, and PubMed. Relevant keywords included ‘HPV’ and ‘vaccine hesitancy’ to ensure thoroughness.

**Review Methods:**

Studies were selected based on their relevance to HPV vaccine hesitancy. Factors contributing to hesitancy were analysed, focusing on antecedents, attributes, and consequences.

**Results:**

The analysis identified key antecedents to HPV vaccine hesitancy, including false cultural beliefs, educational level, knowledge, and vaccine availability. Additional factors were insurance/income, perceived risk, and decision‐making processes. Key attributes impacting hesitancy included media coverage, environmental and community influences, social factors, trust in healthcare systems, efficacy rates, and safety concerns. The consequences of hesitancy were reduced vaccine uptake, increased HPV‐related diseases, and the spread of misinformation.

**Conclusion:**

The study highlights the complex interplay of factors contributing to HPV vaccine hesitancy. Understanding these factors is crucial for addressing the challenges posed by vaccine hesitancy and developing effective interventions.

**Impact:**

Insights from this analysis can guide public health strategies aimed at improving vaccine uptake and reducing HPV‐related diseases. Targeted interventions can address misinformation and enhance trust in vaccines, ultimately leading to better public health outcomes.

**Patient or Public Contribution:**

This concept analysis was conducted without the involvement of patients, service users, caregivers, or members of the public.

## 1 Introduction

The human papillomavirus (HPV) is a prevalent sexually transmitted infection known to cause various cancers, including cervical, anal, and oropharyngeal cancers (Quinlan 2021). HPV vaccines are highly effective in preventing HPV infection and diseases related to HPV infections, especially cervical cancer, which is a significant public health problem, especially in low and middle‐income countries (Shapiro 2022). Most HPV infections (70%–90%) are asymptomatic and will resolve independently within 1–2 years. Persistent infection can cause morbidity and mortality and it is estimated that 4.5%–5.2% of global cancers are explicitly attributed to HPV (Tripathi and Sahu 2022). However, the prevalence and persistence of the HPV vaccine vary by geographical region, sex, age, ethnicity, anatomical location of the infection, weakened immune system, and health behaviours (Wang et al. 2022). The introduction of the HPV vaccine has been a significant public health improvement, promising to reduce the incidence of these cancers globally (Bruni et al. 2021). Despite the vaccine's availability and effectiveness, vaccination rates remain inadequate, posing a significant challenge to public health objectives (Excler, Privor‐Dumm, and Kim 2021). According to the World Health Organization (WHO), vaccine hesitancy refers to a delay in accepting or refusing vaccines despite the availability of vaccination services (Morales‐Campos, Zimet, and Kahn 2023). This incident has been particularly notable in the context of the HPV vaccine. Several factors account for HPV vaccine hesitancy: social, cultural, psychological, individual, and informational (Rezaei n.d.). Understanding the factors contributing to HPV vaccine hesitancy is crucial for developing targeted interventions to increase vaccine uptake and protect public health (Tuckerman, Kaufman, and Danchin 2022). Rodgers's evolutionary concept analysis is an inductive method of analysis which posits that concepts develop over time and are influenced by the context in which they are used (Rodgers 1989). Concepts are continuously evolving, which redefines how their context surrogates, related terms, antecedents, attributes, examples, and consequences are analysed (Rodgers 2000). Such analysis merely indicates a direction for further research. This method is particularly suited for examining HPV vaccine hesitancy, as it allows for the consideration of various contexts and influences that shape the concept.

### 1.1 Background

The introduction of the HPV vaccine has been a significant milestone in public health, offering a means to prevent HPV infections and the consequent development of HPV‐related cancers and other conditions (Illah and Olaitan 2023). The vaccine is recommended for preteens (girls and boys) aged 11–12 years but can be administered as early as nine and up to age 26 (Hofstetter and Schaffer 2021). In some cases, it is recommended for adults up to age 45 who were not vaccinated when they were younger (Hofstetter and Schaffer 2021). The HPV vaccine can significantly reduce the number of HPV‐related cancers, making it a crucial part of cancer prevention efforts worldwide (Giannone et al. 2022). To fully benefit from the vaccine, it is essential to vaccinate as many people as possible (Savulescu 2021). High vaccination rates can lead to widespread immunity, which means fewer cases of high‐risk HPV in the population and a lower overall number of HPV‐related diseases (Williamson 2023). Vaccine hesitancy has appeared as a significant barrier to achieving high HPV vaccination rates (Galagali, Kinikar, and Kumar 2022). This phenomenon, characterised by delay in acceptance or outright refusal of vaccines despite the availability of vaccination services, is influenced by a complex interplay of factors (Dobson 2022). These can include concerns about vaccine safety, misinformation, lack of awareness, cultural beliefs, and distrust in healthcare systems (Cadeddu et al. 2021). Given the critical importance of increasing HPV vaccination rates to prevent HPV‐related diseases, there is a need for a thorough understanding of the factors contributing to HPV vaccine hesitancy (Shapiro 2022). While there is substantial research on vaccine hesitancy in general, there is a need for a focused analysis of HPV vaccine hesitancy. HPV vaccine hesitancy has unique contributing factors, such as misconceptions about the vaccine's safety and efficacy, cultural beliefs, and lack of awareness about HPV and its associated risks (Cadeddu et al. 2021). This paper aims to fill this gap by comprehensively analysing these factors to clarify the attributes, antecedents, and consequences of HPV vaccine hesitancy by employing Rodgers' evolutionary method. This approach helps identify the primary factors and influences that shape the concept over time, providing a thorough understanding that can inform effective public health strategies.

## 2 Methods

This study employed a concept analysis guided by Rodgers' evolutionary method to examine the concept of HPV vaccine hesitancy (Rodgers 2000). Concept analysis is particularly suited to understanding complex, evolving concepts like vaccine hesitancy, as it allows for the exploration of contextual and temporal variations in meaning. Rodgers' evolutionary method was selected because it frames concepts as dynamic, with meanings that shift over time and context. The study design followed six primary activities within Rodgers' method (Rodgers 2000; Tofthagen and Fagerstrøm 2010):

- Identification and naming of the concept: HPV vaccine hesitancy was identified as the central concept of interest, including relevant expressions, such as reluctance, acceptance, and resistance, to capture the full scope of hesitancy.
- Selection of an appropriate sample: A targeted literature review across databases (MEDLINE, CINAHL, PubMed, and PsycINFO) was conducted to retrieve a purposeful sample of studies that illuminate the attributes and dynamics of HPV vaccine hesitancy. Inclusion and exclusion criteria were applied to focus on studies from the past 5 years, in English, and specific to HPV vaccine hesitancy.
- Identification of conceptual elements: The study focused on identifying the concept's core components attributes, antecedents, consequences, surrogates, and related terms that define and contextualise HPV vaccine hesitancy.
- Data analysis of conceptual features: The selected studies were reviewed multiple times to extract data related to HPV vaccine hesitancy's defining characteristics. Data were synthesised according to Rodgers' components, without thematic analysis, to ensure a conceptually focused approach.
- Identification of exemplars: Where appropriate, exemplary cases were identified within the literature to illustrate the application of HPV vaccine hesitancy across different contexts, populations, and health settings.
- Implications and hypotheses for further development: Based on the analysis, implications for understanding and addressing HPV vaccine hesitancy were identified. Potential hypotheses were proposed to guide future research, specifically related to public health interventions and educational efforts.

This concept analysis, grounded in Rodgers' evolutionary approach, allowed for a thorough exploration of HPV vaccine hesitancy as a complex, evolving public health issue.

### 2.1 Identification of Concepts

According to Rodgers (2000), a concept is a word, phrase, or idea that derives meaning from a formal use. This paper aims to establish the meaning of HPV vaccine hesitancy by exploring the various interplay factors contributing to it. HPV vaccine hesitancy refers to uncertainty or indecision regarding vaccination against human papillomavirus. It is characterised by contributing factors such as vaccine efficacy, beliefs, lack of awareness, and associated risks. This definition captures the complex and dynamic nature of HPV hesitancy. According to WHO, vaccine hesitancy is a delay in accepting or refusing vaccines despite the availability of vaccination services (Morales‐Campos, Zimet, and Kahn 2023). This definition applies to all vaccines, including HPV. Also, the American Cancer Society addresses HPV vaccine hesitancy by providing educational resources and emphasising the importance of vaccination for cancer prevention. They highlight barriers such as lack of awareness and misconceptions about the vaccine.

### 2.2 Identification and Selection of Sample and Setting

According to Rodgers (2000), picking a sample and timeframe is essential for a solid study design. The researcher selects the discipline, data sources, literature and period of interest to be included in the study. Rodgers also stresses the need to include a wide range of popular literature in healthcare studies to bridge the gap between professionals' and patients' views. Regarding this study, the area of interest is HPV vaccine hesitancy and its interplay factors. The selected literature comprises articles and published guidelines covering the abovementioned contexts.

### 2.3 Data Collection

According to Rodgers' (2000) evolutionary concept analysis, concept analysis does not require a comprehensive review of all literature but instead focuses on relevant sources that define, clarify, and contextualise the concept. This guided our data collection and selection approach to retrieve a sufficient but manageable number of studies to comprehensively explore HPV vaccine hesitancy. To achieve this, the literature search was conducted across four key databases: MEDLINE, CINAHL, PubMed, and PsycINFO. Search Terms and Strategy: We used targeted keywords to capture multiple facets of HPV vaccine hesitancy, including terms like ‘HPV,’ ‘Human Papillomavirus,’ ‘HPV vaccination,’ ‘HPV immunization,’ ‘HPV prevention,’ ‘vaccine hesitancy,’ ‘vaccine reluctance,’ ‘vaccine acceptance,’ ‘vaccination uptake,’ ‘immunization resistance,’ ‘vaccination compliance,’ and ‘vaccination attitudes.’ The initial search yielded 3662 articles, with 361 duplicates identified and removed.

### 2.4 Inclusion and Exclusion Criteria

Inclusion criteria: To ensure that the data reflected the most recent trends, we limited the selection to studies published in the past 5 years (2019–2024), focused specifically on HPV vaccine hesitancy, and written in English. Exclusion criteria: Studies that addressed vaccines other than HPV, publications over 5 years old, and non‐English articles were excluded. Selection beyond inclusion/exclusion criteria: Beyond the general inclusion/exclusion criteria, we selected studies based on their specific relevance to the conceptual characteristics of HPV vaccine hesitancy. To identify the most conceptually valuable studies, we used the following criteria:

- Relevance to defining conceptual components: Studies were prioritised if they contributed unique insights into attributes, antecedents, consequences, or surrogate terms associated with HPV vaccine hesitancy. This narrowed the pool to studies that provided substantial and nuanced perspectives relevant to the concept's core elements.
- Contextual diversity: We aimed to capture HPV vaccine hesitancy across varied geographic, social, and demographic contexts, selecting studies that addressed hesitancy in populations across different ages, education levels, and cultural settings. This approach helped ensure the concept's robustness by reflecting multiple perspectives.
- Focus on public health and sociocultural factors: Given that HPV vaccine hesitancy is deeply rooted in public health concerns and sociocultural influences, studies were selected if they examined factors like cultural beliefs, misinformation, economic barriers, and healthcare access—components identified as integral to understanding this hesitancy.

**FIGURE 1:**
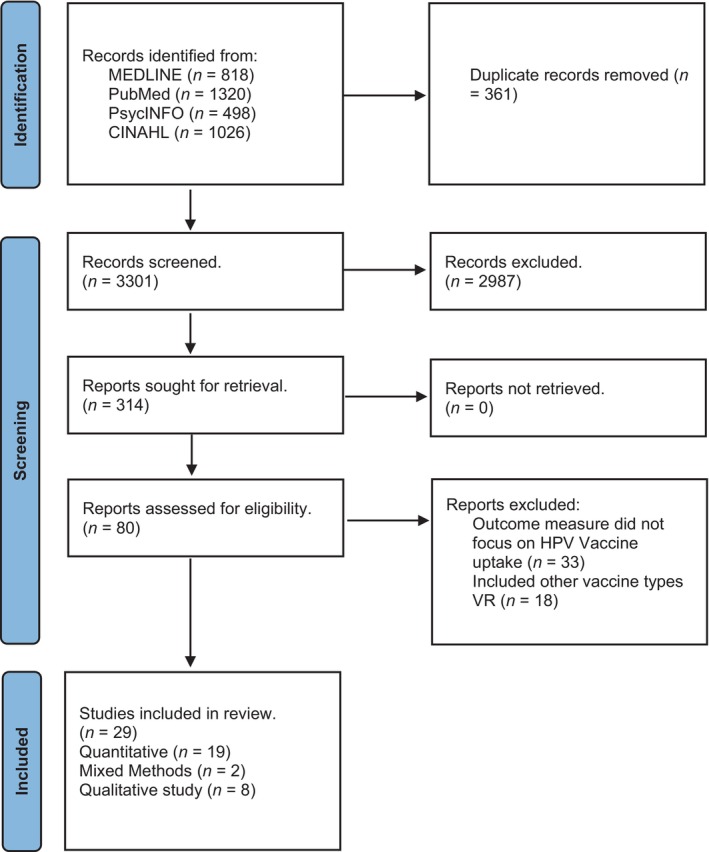
PRISMA diagram showing the number of studies included in the analysis.

Using these targeted criteria, two authors (G.K.K. and E.F.K.) independently screened the articles, ensuring that selected studies were directly relevant to conceptualising HPV vaccine hesitancy. Any disagreements between the two reviewers were resolved through consultation with a third reviewer (R.A.), who served as an arbitrator, ensuring consistency and high relevance. After applying these criteria, 29 articles were finalised for analysis, including 19 quantitative studies, eight qualitative studies, and two mixed‐methods studies. The selection process followed the PRISMA guidelines to ensure transparency, as shown in Figure 1.

### 2.5 Data Extraction and Conflict Resolution

Following study selection, each article was reviewed multiple times by both primary authors (G.K.K. and E.F.K.) to systematically capture the defining characteristics of HPV vaccine hesitancy. Data extraction focused on identifying the attributes, antecedents, and consequences relevant to the concept, as well as any surrogate or related terms.

#### 2.5.1 Extraction Process and Tools

We used a structured set of questions (Table 1), consistent with Rodgers' framework, to guide data extraction. These questions helped both reviewers capture key conceptual aspects of HPV vaccine hesitancy, ensuring that data extraction focused on conceptual clarity rather than thematic trends, as per the principles of concept analysis. Examples of questions included:

- *What are the characteristics and qualities of HPV vaccine hesitancy?*
- *What contextual factors precede the expression of this hesitancy?*
- *What consequences result from HPV vaccine hesitancy within a population?*

**TABLE 1:** Questions used for data extraction.

Term	Questions
Surrogate terms	Is the researcher using a similar word for the concept?/Are there other ways of expressing the concept?
Related terms	Is this concept related to another concept?
Antecedents	What contextual factors precede the expression of this hesitancy?
Attributes	What are the characteristics or qualities of this concept?
Examples	Do we have concrete examples of this concept that have been described in detail?
Consequences	What consequences result from HPV vaccine hesitancy within a population

Inter‐rater reliability and consensus process: To address consistency and minimise subjective bias: Independent coding and comparison: Each reviewer independently extracted data and coded conceptual elements, and their findings were then compared. Although kappa statistics were not employed, a consensus‐based approach was used to ensure inter‐rater reliability. Any discrepancies in extracted data or coding were openly discussed to reach consensus. Conflict resolution: When consensus was not readily achieved, a third reviewer (R.A.) reviewed the conflicting data points to provide a final decision, which was incorporated into the analysis. This rigorous approach ensured that only conceptually relevant data were included and consistently applied across all studies.

### 2.6 Synthesis of Conceptual Attributes

The analysis did not employ thematic categorisation, as concept analysis focuses on defining a concept rather than deriving themes. Instead, we organised the findings according to conceptual components: Attributes, Antecedents, and Consequences: We synthesised the extracted data into these three main components. Where differences in categorisation arose, these were resolved collaboratively amongst the reviewers, focusing on emerging trends and areas for further research. This approach allowed us to comprehensively represent HPV vaccine hesitancy and its defining aspects without imposing thematic structures. This detailed approach ensures a high standard of methodological rigour and conceptual transparency, allowing the study to reflect the complex nature of HPV vaccine hesitancy thoroughly and accurately in line with Rodgers' evolutionary concept analysis framework.

## 3 Results

After initial screening with titles and abstracts and applying inclusion and exclusion criteria during full‐text reading, only 29 articles were eligible for the final analysis. Nineteen were quantitative, eight were qualitative, and two were mixed‐method studies, and they were published between 2019 and 2024.

### 3.1 Surrogate Terms

Surrogate terms are synonyms or alternative expressions that convey similar meanings to the concept of vaccine hesitancy, allowing for nuanced understanding and interpretation (Rodgers 2000) (Table 2). In this study, several surrogate terms were identified:

- Vaccine reluctance: This term refers to mild uncertainty or hesitation about vaccination, where individuals may be hesitant but remain open to eventual acceptance (Bonanni et al. 2018; Chen, Chen, et al. 2024; Chen, Wang, et al. 2024; Elhaddadi et al. 2024; Hansen, Schmidtblaicher, and Brewer 2020; Howard 2023; Khosa et al. 2022; Marshall et al. 2019; Montalti et al. 2024; Redd et al. 2024; Rositch et al. 2022; Santhanes et al. 2018; Shato et al. 2023; Siu, Fung, and Leung 2019; Zhang et al. 2021).
- Immunisation hesitation: This term highlights the delay or uncertainty in deciding to receive a vaccine, capturing the psychological aspect of hesitancy without outright refusal (Wilson et al. 2023).
- Vaccination resistance: This stronger term describes an active or firm refusal to vaccinate, often influenced by established beliefs or distrust toward vaccination (Alsanafi, Salim, and Sallam 2023; Hansen, Schmidtblaicher, and Brewer 2020).
- Vaccine scepticism: This term emphasises doubt or mistrust toward vaccines and can lead to reluctance or resistance if not addressed (Dib et al. 2022; O'Marr et al. 2023; Walker, Owens, and Zimet 2020).

**TABLE 2:** Surrogate terms and related concepts.

Category	Term	Reference
Surrogate terms	Vaccine reluctance	(Bonanni et al. 2018; Chen, Chen, et al. 2024; Chen, Wang, et al. 2024; Elhaddadi et al. 2024; Hansen, Schmidtblaicher, and Brewer 2020; Howard 2023; Khosa et al. 2022; Marshall et al. 2019; Montalti et al. 2024; Redd et al. 2024; Rositch et al. 2022; Santhanes et al. 2018; Shato et al. 2023; Siu, Fung, and Leung 2019; Zhang et al. 2021)
	Immunisation hesitation	(Wilson et al. 2023)
	Vaccine scepticism	(Dib et al. 2022; O'Marr et al. 2023; Walker, Owens, and Zimet 2020)
	Vaccination resistance	(Alsanafi, Salim, and Sallam 2023; Hansen, Schmidtblaicher, and Brewer 2020)
Related terms	Vaccination indecision	(Beavis et al. 2022; Dib et al. 2022; Hansen, Schmidtblaicher, and Brewer 2020; Rositch et al. 2022; Santhanes et al. 2018; Wilson et al. 2023)
	Immunisation avoidance	(Pomares et al. 2020; Wilson et al. 2023)

Each of these surrogate terms aligns closely with vaccine hesitancy but reflects variations in the degree of hesitation or resistance. By identifying these distinctions, we aim to provide a more comprehensive understanding of hesitancy‐related behaviours.

#### 3.1.1 Related Terms

Related terms are concepts associated with vaccine hesitancy but that do not fully share its attributes or connotations (Rodgers 2000) (Table 2). Related terms identified in this study include:

- Vaccination indecision: This concept indicates a state of uncertainty in which individuals are undecided about vaccinating, though they may not necessarily distrust vaccines (Beavis et al. 2022; Dib et al. 2022; Hansen, Schmidtblaicher, and Brewer 2020; Rositch et al. 2022; Santhanes et al. 2018; Wilson et al. 2023).
- Vaccination avoidance: This term describes an intentional choice to avoid vaccination, often due to low perceived risk of disease or prioritisation of other health behaviours (Pomares et al. 2020; Wilson et al. 2023).

By distinguishing surrogate terms from related terms, we provide a clearer framework for understanding various manifestations of vaccine hesitancy and its nuances. These distinctions help refine the conceptual boundaries of vaccine hesitancy in the context of HPV vaccination.

### 3.2 Antecedents

According to Rodgers (2000), antecedents are events or phenomena that precede and are related to the concept Table 3. In this study, antecedents contributing to individual uncertainty about getting the HPV vaccine include:

1. *False cultural beliefs*: Most of the beliefs are misconceptions and misinformation rooted in their culture, and these may also stem from historical and social contexts that shape community attitudes toward vaccines. Several studies highlight that some communities believe that the HPV vaccine encourages promiscuity despite no scientific evidence supporting this claim (Bonanni et al. 2018; Dib et al. 2022; Hansen, Schmidtblaicher, and Brewer 2020; Howard 2023; Khosa et al. 2022; Marshall et al. 2019; Montalti et al. 2024; O'Marr et al. 2023; Redd et al. 2024). Additionally, myths about the vaccine causing infertility to persist in specific cultural contexts, further deterring vaccination efforts (Santhanes et al. 2018; Siu, Fung, and Leung 2019; Srivastava et al. 2023; Wilson et al. 2023; Wiyeh et al. 2019). The persistence of false cultural beliefs poses a substantial barrier to achieving optimal HPV vaccination rates.
2. *Education level/knowledge/awareness*: Education level, knowledge, and awareness about HPV and the HPV vaccine significantly influence vaccination decisions. Study findings indicate that individuals with higher education levels are more likely to understand the benefits and safety of the HPV vaccine, leading to higher vaccination rates (Alsanafi, Salim, and Sallam 2023; Alshamlan et al. 2024; Beavis et al. 2022; Bonanni et al. 2018; Chen, Chen, et al. 2024). Conversely, low education levels are linked to greater susceptibility to misinformation and false beliefs about the vaccine (Chen, Wang, et al. 2024; Dib et al. 2022; Elhaddadi et al. 2024; Howard 2023; Marshall et al. 2019; Montalti et al. 2024). Likewise, in other study findings, parents' well‐informed about the vaccine's role in preventing cervical cancer were more likely to vaccinate their children (Pomares et al. 2020; Redd et al. 2024; Rositch et al. 2022; Santhanes et al. 2018; Siu, Fung, and Leung 2019). On the other hand, a lack of awareness about the vaccine and its benefits contributed significantly to hesitancy (Wemrell and Gunnarsson 2022; Wilson et al. 2023; Wiyeh et al. 2019; Zhang et al. 2021).
3. *Family medical history*: Family medical history can significantly impact HPV vaccine acceptance. Other studies have found that individuals from families with a history of adverse vaccine reactions are more likely to exhibit hesitancy toward the HPV vaccine (Alshamlan et al. 2024; Chen, Chen, et al. 2024; Chen, Wang, et al. 2024).
4. *Income/insurance coverage/cost*: Study findings show that individuals with health insurance that covers the HPV vaccine are more likely to vaccinate their children compared to those without such coverage (Chen, Chen, et al. 2024; Howard 2023; Srivastava et al. 2023). Additionally, lower income levels are associated with higher rates of vaccine hesitancy, often due to concerns about the cost of the vaccine and access to healthcare services (Alsanafi, Salim, and Sallam 2023; Elhaddadi et al. 2024; Wemrell and Gunnarsson 2022; Zakhour et al. 2023). Study findings found that out‐of‐pocket costs were a significant limitation for parents considering the HPV vaccine for their children (Rositch et al. 2022; Santhanes et al. 2018).
5. *Vaccine availability/constraints*: Study findings revealed that individuals living in rural areas had difficulty accessing the various hospitals for the vaccine due to distance and limited clinic hours (Alsanafi, Salim, and Sallam 2023; Khosa et al. 2022). Other studies also indicate that a shortage of vaccines in various facilities and timely vaccines were the deterrents to vaccination (Alshamlan et al. 2024; Howard 2023; Montalti et al. 2024; Wiyeh et al. 2019; Zakhour et al. 2023).
6. *Age group*: Different age groups may have varying levels of awareness, perceived risk, and attitudes towards vaccination. These studies indicate that parents' knowledge and attitudes significantly impact vaccination rates in this group (Alsanafi, Salim, and Sallam 2023; Alshamlan et al. 2024; Elenwo et al. 2023). Other findings from similar studies also revealed that older adolescents and young adults are more likely to be influenced by peer opinions and their perceptions of risk and benefit (Montalti et al. 2024; Shato et al. 2023; Srivastava et al. 2023). Other findings also found that young adults who perceived themselves as being at lower risk for HPV were less likely to seek vaccination (Wemrell and Gunnarsson 2022; Wiener et al. 2020).
7. *Vaccine history*: The study findings of the following studies show that individuals within the community who have had personal adverse effects and an adverse history of the vaccine deter them from getting vaccinated (Chen, Wang, et al. 2024; Redd et al. 2024; Shato et al. 2023).
8. *Perceived risk*: The study findings reveal that individuals who perceive themselves at high risk of contracting HPV are more likely to accept the vaccine (Beavis et al. 2022; Chen, Chen, et al. 2024; Elenwo et al. 2023; Howard 2023; Khosa et al. 2022). Also, concerns about vaccine safety and potential side effects contribute to vaccine hesitancy (Montalti et al. 2024; O'Marr et al. 2023; Siu, Fung, and Leung 2019; Walker, Owens, and Zimet 2020; Wemrell and Gunnarsson 2022).
9. *Decision‐making*: The decision‐making process involves various factors, including individual autonomy, parental influence, and healthcare recommendations. This is a significant complex aspect of vaccination. Studies indicate that individuals actively involved in the decision‐making process and who receive recommendations from trusted healthcare providers are more likely to accept the HPV vaccine (Alshamlan et al. 2024; Beavis et al. 2022; Dib et al. 2022). Parental decision‐making heavily influences HPV vaccine uptake amongst adolescents (O'Marr et al. 2023; Pomares et al. 2020; Siu, Fung, and Leung 2019; Wemrell and Gunnarsson 2022).
10. *Self‐affirmations*: Findings reveal that self‐affirmations can reduce resistance and increase openness to health interventions (O'Marr et al. 2023). Additionally, individuals who engage in self‐affirmations before receiving health messages about the HPV vaccine show higher acceptance rates.
11. *Cognitive biases*: Cognitive biases, such as confirmation bias, availability heuristics, and optimism bias, can influence individuals' perceptions and decisions about vaccination. Study findings show that confirmation bias may lead individuals to seek information supporting their beliefs against vaccination. At the same time, availability strategy can cause them to overestimate the likelihood of adverse events based on recent or memorable cases (Dib et al. 2022; Pomares et al. 2020; Wemrell and Gunnarsson 2022).

**TABLE 3:** Antecedents, attributes, and consequences of HPV vaccine hesitancy.

Category	Term	References
Antecedents	False cultural belief	(Bonanni et al. 2018; Dib et al. 2022; Hansen, Schmidtblaicher, and Brewer 2020; Howard 2023; Khosa et al. 2022; Marshall et al. 2019; Montalti et al. 2024; O'Marr et al. 2023; Redd et al. 2024; Santhanes et al. 2018; Siu, Fung, and Leung 2019; Srivastava et al. 2023; Wilson et al. 2023; Wiyeh et al. 2019)
	Education level/knowledge/level of awareness	(Alsanafi, Salim, and Sallam 2023; Alshamlan et al. 2024; Beavis et al. 2022; Bonanni et al. 2018; Chen, Chen, et al. 2024; Chen, Wang, et al. 2024; Dib et al. 2022; Elhaddadi et al. 2024; Howard 2023; Marshall et al. 2019; Montalti et al. 2024; Pomares et al. 2020; Redd et al. 2024; Rositch et al. 2022; Santhanes et al. 2018; Siu, Fung, and Leung 2019; Wemrell and Gunnarsson 2022; Wilson et al. 2023; Wiyeh et al. 2019; Zhang et al. 2021)
	Family medical history	(Alshamlan et al. 2024; Chen, Chen, et al. 2024; Chen, Wang, et al. 2024)
	Income/insurance coverage/cost	(Alsanafi, Salim, and Sallam 2023; Chen, Chen, et al. 2024; Elhaddadi et al. 2024; Howard 2023; Rositch et al. 2022; Santhanes et al. 2018; Srivastava et al. 2023; Wemrell and Gunnarsson 2022; Zakhour et al. 2023)
	Vaccine availability/constraints	(Alsanafi, Salim, and Sallam 2023; Alshamlan et al. 2024; Howard 2023; Khosa et al. 2022; Montalti et al. 2024; Wiyeh et al. 2019; Zakhour et al. 2023)
	Age group	(Alsanafi, Salim, and Sallam 2023; Alshamlan et al. 2024; Elenwo et al. 2023; Montalti et al. 2024; Shato et al. 2023; Srivastava et al. 2023; Wemrell and Gunnarsson 2022; Wiener et al. 2020)
	Vaccine history	(Chen, Wang, et al. 2024; Redd et al. 2024; Shato et al. 2023)
	Perceived risk	(Beavis et al. 2022; Chen, Chen, et al. 2024; Elenwo et al. 2023; Howard 2023; Khosa et al. 2022; Montalti et al. 2024; O'Marr et al. 2023; Siu, Fung, and Leung 2019; Walker, Owens, and Zimet 2020; Wemrell and Gunnarsson 2022)
	Decision making	(Alshamlan et al. 2024; Beavis et al. 2022; Dib et al. 2022; O'Marr et al. 2023; Pomares et al. 2020; Siu, Fung, and Leung 2019; Wemrell and Gunnarsson 2022)
	Self‐affirmations	(O'Marr et al. 2023)
	Cognitive biases	(Dib et al. 2022; Pomares et al. 2020; Wemrell and Gunnarsson 2022)
Attributes	Media coverage/campaign/social media influence	(Alsanafi, Salim, and Sallam 2023; Beavis et al. 2022; Dib et al. 2022; Hansen, Schmidtblaicher, and Brewer 2020; Marshall et al. 2019; Montalti et al. 2024; Redd et al. 2024; Santhanes et al. 2018; Siu, Fung, and Leung 2019; Thompson et al. 2017; Walker, Owens, and Zimet 2020; Zakhour et al. 2023; Zhang et al. 2021)
	Environmental/community/social factors	(Beavis et al. 2022; Chen, Chen, et al. 2024; Montalti et al. 2024; Rositch et al. 2022; Wiener et al. 2020; Wilson et al. 2023; Zhang et al. 2021)
	Trust in healthcare systems	(Beavis et al. 2022; Chen, Wang, et al. 2024; Dib et al. 2022; Siu, Fung, and Leung 2019; Walker, Owens, and Zimet 2020; Wemrell and Gunnarsson 2022; Wilson et al. 2023)
	Social and geopolitical influence	(Dib et al. 2022; Howard 2023; Marshall et al. 2019; Montalti et al. 2024; Wilson et al. 2023)
	Efficacy rate	(Beavis et al. 2022; Bonanni et al. 2018; Dib et al. 2022; Elenwo et al. 2023; Khosa et al. 2022; Redd et al. 2024; Santhanes et al. 2018; Walker, Owens, and Zimet 2020; Zakhour et al. 2023; Zhang et al. 2021)

	Vaccine timing/program structure	(Dib et al. 2022; Hansen, Schmidtblaicher, and Brewer 2020; Khosa et al. 2022; Montalti et al. 2024; O'Marr et al. 2023; Srivastava et al. 2023; Wiener et al. 2020; Wiyeh et al. 2019)
	Complacency	(Alsanafi, Salim, and Sallam 2023; Chen, Chen, et al. 2024; Khosa et al. 2022; Siu, Fung, and Leung 2019)
	Confidence	(Chen, Chen, et al. 2024; Khosa et al. 2022; Siu, Fung, and Leung 2019; Srivastava et al. 2023; Walker, Owens, and Zimet 2020)
	Convenience	(Chen, Chen, et al. 2024; Howard 2023; Siu, Fung, and Leung 2019)
	Safety concerns	(Alshamlan et al. 2024; Beavis et al. 2022; Bonanni et al. 2018; Chen, Wang, et al. 2024; Dib et al. 2022; Elhaddadi et al. 2024; Howard 2023; Khosa et al. 2022; Marshall et al. 2019; Montalti et al. 2024; Redd et al. 2024; Rositch et al. 2022; Shato et al. 2023; Siu, Fung, and Leung 2019; Wilson et al. 2023; Zakhour et al. 2023)
	Perceived promiscuity	(Alshamlan et al. 2024; Siu, Fung, and Leung 2019)
Immunogenicity	(Bonanni et al. 2018; Wiyeh et al. 2019)
Consequences	Reduced vaccine uptake	(Alsanafi, Salim, and Sallam 2023; Alshamlan et al. 2024; Beavis et al. 2022; Chen, Wang, et al. 2024; Dib et al. 2022; Elhaddadi et al. 2024; Hansen, Schmidtblaicher, and Brewer 2020; Khosa et al. 2022; Marshall et al. 2019; Pomares et al. 2020; Rositch et al. 2022; Santhanes et al. 2018; Shato et al. 2023; Wemrell and Gunnarsson 2022; Wilson et al. 2023; Wiyeh et al. 2019; Zakhour et al. 2023)
	Missed early vaccination opportunities	(Hansen, Schmidtblaicher, and Brewer 2020; Santhanes et al. 2018)
	Increase in HPV‐related cancers	(Alsanafi, Salim, and Sallam 2023; Chen, Wang, et al. 2024; Dib et al. 2022; Elhaddadi et al. 2024; Pomares et al. 2020; Rositch et al. 2022; Santhanes et al. 2018; Wemrell and Gunnarsson 2022; Wilson et al. 2023)
	Spread of misinformation	(Beavis et al. 2022; Bonanni et al. 2018; Elhaddadi et al. 2024; Walker, Owens, and Zimet 2020; Wilson et al. 2023; Wiyeh et al. 2019)
	Strained healthcare relationships	(Beavis et al. 2022)
	Negative vaccination attitudes	(O'Marr et al. 2023; Wilson et al. 2023)

### 3.3 Attributes

Attributes are the characteristics or qualities of a concept and constitute its actual definition (Rodgers 2000). They enable the identification of situations that fall under the concept of study and help distinguish the true meaning of the concept from a mere dictionary definition Table 3. In this study, attributes for HPV vaccine hesitancy include:

1. *Information source/campaign*: The source of information and campaign about vaccines play a significant role in vaccination because of the origin and nature of information individuals receive about vaccines. The study findings show that exposure to content disregarding vaccines on social media links and misinformation from less reliable sources relates to increased vaccine hesitancy (Alsanafi, Salim, and Sallam 2023; Beavis et al. 2022; Dib et al. 2022; Hansen, Schmidtblaicher, and Brewer 2020; Marshall et al. 2019; Montalti et al. 2024; Redd et al. 2024; Santhanes et al. 2018; Siu, Fung, and Leung 2019; Walker, Owens, and Zimet 2020; Zakhour et al. 2023; Zhang et al. 2021).
2. *Environmental/community/social factors*: These attributes involves the broader context in which individuals live and make decisions about vaccination. It includes the influence of community norms, social networks, and the environment. People are often influenced by the opinions and behaviours of those around them, including family, friends, and community leaders. In communities and certain social circles, mistrust of vaccines may be widespread due to historical experiences or cultural beliefs, leading to higher hesitancy (Beavis et al. 2022; Chen, Chen, et al. 2024; Montalti et al. 2024; Rositch et al. 2022; Wiener et al. 2020; Wilson et al. 2023; Zhang et al. 2021).
3. *Trust in the healthcare system*: Trust in the healthcare system is a fundamental attribute of vaccine hesitancy. It refers to individuals' confidence in medical institutions and professionals. Other studies have shown that people with low trust in the healthcare system are prone to hesitate about vaccines, and trust is influenced by factors such as past experiences, perceived competence, and transparency of healthcare providers (Beavis et al. 2022; Chen, Wang, et al. 2024; Dib et al. 2022; Siu, Fung, and Leung 2019; Walker, Owens, and Zimet 2020; Wemrell and Gunnarsson 2022; Wilson et al. 2023).
4. *Socio/political influence*: This attribute consists of a broader context of the socio and political influences on individual behaviour towards vaccination. Anti‐vaccine movements, often driven by sociopolitical systems, spread misinformation and create public fear and mistrust (Dib et al. 2022; Marshall et al. 2019; Montalti et al. 2024). Vaccination efforts are often disrupted in regions affected by war or civil unrest, leading to lower coverage and outbreaks of vaccine‐preventable diseases (Howard 2023; Wilson et al. 2023).
5. *Efficacy rate*: Misinformation and a lack of understanding about efficacy rates can lead to vaccine hesitancy. Individuals may underestimate the vaccine's benefits if they do not fully understand its protection against multiple HPV strains (Beavis et al. 2022; Bonanni et al. 2018; Dib et al. 2022; Elenwo et al. 2023; Khosa et al. 2022). Other study findings show that people understand that the HPV vaccine offers high efficacy in preventing severe health conditions like cervical cancer, and they are more likely to perceive it as a valuable preventive measure (Redd et al. 2024; Santhanes et al. 2018; Walker, Owens, and Zimet 2020; Zakhour et al. 2023; Zhang et al. 2021).
6. *Vaccine timing/program structure*: Study show that administering the vaccine before individuals become sexually active prevents HPV infections (Dib et al. 2022). Other study findings also discussed the importance of adhering to the recommended vaccine schedule (Hansen, Schmidtblaicher, and Brewer 2020). The HPV vaccine is typically administered in a two‐ or three‐dose schedule, depending on the age at the start of vaccination. The success of well‐designed vaccination programs hinges on the importance of community engagement and education. These programs must be accessible and convenient for the target population while also educating the public about the importance of timely HPV vaccination (Khosa et al. 2022; Montalti et al. 2024; O'Marr et al. 2023; Srivastava et al. 2023; Wiener et al. 2020; Wiyeh et al. 2019).
7. *Complacency*: Complacency often stems from a low perception of risk associated with HPV infection. Studies highlight that individuals who do not perceive themselves or their children to be at risk of contracting HPV are less likely to prioritise vaccination, and many individuals are unaware of the link between HPV and various cancers (Alsanafi, Salim, and Sallam 2023; Chen, Chen, et al. 2024). Study findings show how recommendations from trusted healthcare providers can significantly influence vaccine acceptance and explore how social and cultural factors contribute to complacency (Khosa et al. 2022; Siu, Fung, and Leung 2019).
8. *Confidence*: Findings from Chen and colleagues found that when healthcare providers take the time to explain the benefits and safety of the HPV vaccine, patients are more likely to trust the vaccine and proceed with vaccination (Chen, Chen, et al. 2024). Other studies shown how well‐designed educational programs that address common concerns and provide clear information can significantly increase confidence in the HPV vaccine, leading to higher vaccination rates (Khosa et al. 2022; Walker, Owens, and Zimet 2020). Other studies highlight the impact of media on public perception and social and cultural factors that influence confidence in the HPV vaccine (Siu, Fung, and Leung 2019; Srivastava et al. 2023).
9. *Convenience*: Convenience in accessing vaccination services is fundamental. Studies have found that these programs significantly increase vaccination rates by making the vaccine accessible to students during school hours and emphasising the importance of a straightforward vaccination process, reducing the need for additional appointments and travel (Howard 2023; Siu, Fung, and Leung 2019). Chen and colleagues demonstrate that employers who provide on‐site vaccination clinics or allow employees time off to get vaccinated see higher participation rates and that vaccination services are easily accessible (Chen, Chen, et al. 2024).
10. *Safety concerns*: Findings highlight fears about immediate adverse reactions, such as pain at the injection site, fever, and fainting, which can deter individuals from vaccinating (Alshamlan et al. 2024; Beavis et al. 2022). Addressing these concerns through clear communication about most side effects' common, mild, and temporary nature can alleviate fears and increase acceptance. Other study findings reveal how uncertainty about long‐term effects can contribute to vaccine hesitancy (Bonanni et al. 2018; Chen, Wang, et al. 2024; Zakhour et al. 2023). Other study findings show the role of social media in propagating false information about vaccines, such as unfounded claims linking the HPV vaccine to infertility or other severe conditions (Dib et al. 2022; Elhaddadi et al. 2024; Wilson et al. 2023). Other findings emphasise that trusted healthcare providers can effectively communicate the safety profile of the HPV vaccine, answer patients' questions, and provide reassurance (Howard 2023; Khosa et al. 2022). These studies discuss how dramatised media reports of rare adverse events excessively affect public opinion (Marshall et al. 2019; Montalti et al. 2024). Also, findings from other studies indicate the effectiveness of public health campaigns in educating the public about the rigorous safety testing vaccines undergo and the continuous monitoring for adverse events (Redd et al. 2024; Rositch et al. 2022). Other similar studies also note that public confidence can be encouraged by highlighting the severe regulatory processes that vaccines must pass before approval, including clinical trials and post‐marketing investigation (Shato et al. 2023; Siu, Fung, and Leung 2019).
11. *Perceived promiscuity*: Findings from Siu and Colleagues found that when healthcare providers explain the importance of the HPV vaccine in preventing severe health issues, parents are more likely to overcome their concerns about perceived promiscuity and consent to vaccination (Siu, Fung, and Leung 2019). Other study findings highlight successful campaigns emphasising cancer prevention, effectively changing perceptions, and increasing vaccine uptake (Alshamlan et al. 2024).
12. *Immunogenicity*: Study findings highlight how the strong immune responses observed in clinical trials have led to the inclusion of HPV vaccines in national immunisation programs worldwide (Bonanni et al. 2018). Other study findings discuss how monitoring antibody levels over time has provided evidence for the potential need for booster doses in specific populations to maintain high levels of protection (Wiyeh et al. 2019).

### 3.4 Consequences

Consequences are events that result from the use of a concept. HPV vaccine hesitancy has led to several significant outcomes Table 3. In this study, the consequences of HPV vaccine hesitancy include:

1. *Reduced vaccine uptake*: Misinformation, cultural and religious beliefs, socioeconomic barriers, demographics, and communication challenges all play significant roles in reducing vaccine uptake (Alsanafi, Salim, and Sallam 2023; Alshamlan et al. 2024; Beavis et al. 2022; Chen, Wang, et al. 2024; Dib et al. 2022; Elhaddadi et al. 2024; Hansen, Schmidtblaicher, and Brewer 2020; Khosa et al. 2022; Marshall et al. 2019; Pomares et al. 2020; Rositch et al. 2022; Santhanes et al. 2018; Shato et al. 2023; Wemrell and Gunnarsson 2022; Wilson et al. 2023; Wiyeh et al. 2019; Zakhour et al. 2023).
2. *Missed early vaccination*: Myths and misinformation about the HPV vaccine's safety and necessity have led to confusion and fear amongst parents. As a result, many parents postpone or skip early vaccination, believing it might be harmful or unnecessary (Hansen, Schmidtblaicher, and Brewer 2020; Santhanes et al. 2018).
3. *Increase in HPV‐related cancers*: Studies have highlighted that misinformation about the HPV vaccine has led to increased vaccine hesitancy and subsequently higher rates of HPV‐related diseases (Alsanafi, Salim, and Sallam 2023; Rositch et al. 2022). Misinformation influences socioeconomic and educational disparities, along with demographic factors, resulting in uneven vaccine uptake and higher disease rates in specific populations (Chen, Wang, et al. 2024; Dib et al. 2022). Social dynamics and cultural beliefs further contribute to vaccine hesitancy, leading to higher incidences of HPV‐related diseases (Wemrell and Gunnarsson 2022; Wilson et al. 2023). Additionally, misinformation and increased healthcare costs are associated with higher rates of HPV‐related diseases due to reduced vaccine uptake (Elhaddadi et al. 2024; Pomares et al. 2020; Santhanes et al. 2018).
4. *Spread of misinformation*: The spread of misinformation through various channels, including social media and community networks, contributes significantly to vaccine hesitancy (Beavis et al. 2022; Bonanni et al. 2018). Findings reveal the psychological impact misinformation has had on increasing fears and misconceptions about the safety and efficacy of the HPV vaccine (Elhaddadi et al. 2024; Walker, Owens, and Zimet 2020). Misinformation also interacts with cultural and religious beliefs to deepen vaccine hesitancy in specific communities (Wilson et al. 2023; Wiyeh et al. 2019).
5. *Strained healthcare relationships*: A study by Beavis and colleagues identifies that misinformation spread by the concept paper has caused patients to question the credibility of healthcare providers (Beavis et al. 2022). This loss of trust has led to increased vaccine hesitancy, as patients are less likely to accept recommendations from healthcare providers, they do not fully trust.
6. *Negative vaccination attitude*: O'Marr et al. (2023) focus on the social and psychological barriers that lead to reluctance in accepting the HPV vaccine. They emphasise that fear of adverse effects and the spread of misinformation through social media have worsened vaccine hesitancy. Wilson et al. (2023) delve into specific misconceptions surrounding the HPV vaccine, particularly the unfounded fears that it may encourage risky sexual behaviour or lead to infertility.

### 3.5 Definition of Concept

HPV vaccine hesitancy is the reluctance or refusal to vaccinate against the human papillomavirus despite the availability of vaccination services. It is influenced by factors such as false cultural beliefs, education level, perceived risk, and trust in healthcare systems. This hesitancy is characterised by safety concerns, media influence, and social factors, leading to reduced vaccine uptake, increased HPV‐related cancers, and the spread of misinformation.

## 4 Discussion

Our study has identified a range of antecedents, attributes, and consequences contributing to HPV vaccine hesitancy and underscores the need for targeted, multifactorial interventions. Deep‐rooted cultural beliefs are a significant contributor, as misconceptions linking the vaccine to promiscuity or infertility can deter individuals from getting vaccinated. These beliefs are often spread through community norms and misinformation, making culturally sensitive educational campaigns critical to counteracting them (Wiyeh et al. 2019). Additionally, myths and misinformation surrounding the vaccine perpetuate hesitancy, leading some individuals to delay or refuse vaccination based on unfounded fears. This hesitancy can result in missed early vaccination opportunities, which are critical for ensuring optimal immune response and long‐term protection against HPV (Hansen, Schmidtblaicher, and Brewer 2020; Santhanes et al. 2018; Walker, Owens, and Zimet 2020; Wilson et al. 2023; Wiyeh et al. 2019).

Socioeconomic factors, such as lower education levels, also correlate with increased vaccine hesitancy. Individuals with limited health literacy may struggle to understand the benefits and safety of the HPV vaccine (Marshall et al. 2019). Therefore, enhancing health education and providing clear, accessible information is essential to improving vaccine uptake (O'Marr et al. 2023). Awareness of the HPV vaccine and its benefits is critical for vaccine acceptance, as misinformation and lack of knowledge can lead to hesitancy (Bonanni et al. 2018). Public health campaigns that raise awareness and correct false information are necessary to combat these issues (Srivastava et al. 2023). Personal and familial medical history can also influence vaccine decisions; for example, families with a history of adverse reactions to vaccines or other medical treatments may be more hesitant (Chen, Chen, et al. 2024). Healthcare providers must address these concerns through personalised consultations to build trust and improve vaccine acceptance (Alshamlan et al. 2024). Economic barriers and healthcare access significantly impact vaccine uptake. Individuals with lower income or inadequate insurance coverage often find the vaccine cost prohibitive. Policies to provide free or subsidised vaccines can improve access and reduce hesitancy, particularly in low‐income and underserved communities (Zakhour et al. 2023). Limited vaccine availability and logistical constraints, such as transportation challenges and limited clinic hours, can further hinder access (Khosa et al. 2022). Ensuring widespread availability and convenient access points is essential to increase vaccination rates across diverse socioeconomic groups.

HPV vaccine hesitancy varies across different age groups and demographics. Parents may be hesitant to vaccinate their children due to concerns about safety and side effects (Wiener et al. 2020). Tailored communication strategies are therefore needed to address the specific concerns of each demographic. Negative past experiences with vaccines or observed adverse reactions can also contribute to hesitancy (Shato et al. 2023). Building positive experiences through safe and effective vaccination programs is essential to counteract these biases. Individual perceptions of risk—both of contracting HPV and of vaccine‐related side effects influence decisions to vaccinate. Cognitive biases, such as overestimating vaccination risks and underestimating HPV risks, can skew this decision‐making process (Pomares et al. 2020). Addressing these cognitive biases with targeted information can improve decision‐making (Wemrell and Gunnarsson 2022). Information sources, including media coverage and social media, play a powerful role in shaping public perception of the HPV vaccine. Sensationalist reporting on adverse events can increase fear and hesitancy, while balanced and factual coverage can enhance acceptance (Beavis et al. 2022). Public health authorities should engage with media outlets to promote accurate messaging about the vaccine. Community norms and social influences significantly shape vaccination behaviours, with individuals more likely to vaccinate if it aligns with community expectations (Rositch et al. 2022). Community‐based interventions and peer education programs can help shift norms towards vaccine acceptance, even in communities with prevalent vaccine scepticism (Siu, Fung, and Leung 2019). Trust in healthcare providers and the healthcare system is also crucial for vaccine acceptance, as it shapes individuals' willingness to follow medical advice and engage in preventive care (Chen, Wang, et al. 2024). Historical instances of unethical medical practices, particularly involving minority populations, have fostered a deep‐seated distrust of healthcare systems, impacting vaccine acceptance and public health efforts. This distrust can create hesitancy around vaccines, as communities fear potential exploitation or lack of transparency (Wilson et al. 2023).

Additionally, socioeconomic disparities further compound these issues. Limited access to quality healthcare and financial constraints can exacerbate feelings of alienation and scepticism toward medical institutions, especially amongst underserved communities. Addressing this requires transparent, respectful, and culturally competent communication, where healthcare providers actively engage with communities, acknowledge past injustices, and emphasise equitable care to rebuild trust and promote vaccination in vulnerable populations. Access to healthcare and cultural differences vary significantly across regions, particularly between high‐income and low‐ to middle‐income countries, influencing public attitudes and rates of HPV vaccine uptake. In low‐income countries, barriers such as limited healthcare infrastructure, high vaccine costs, and inadequate transportation options often prevent widespread vaccination (Zakhour et al. 2023; Khosa et al. 2022). These logistical challenges can lead to higher rates of vaccine hesitancy, as access issues overshadow vaccine benefits. In contrast, high‐income countries often have better healthcare systems and vaccine accessibility, but vaccine hesitancy in these areas is more commonly influenced by misinformation, low perceived risk, and cultural misconceptions. For instance, concerns about vaccine safety, fueled by media coverage or historical mistrust in healthcare, may deter people from vaccination in high‐resource settings. Recognising these geographic and contextual variabilities is critical for developing public health interventions that are tailored to local needs, ensuring strategies that address both physical access in low‐income settings and informational and cultural barriers in wealthier regions.

Perceptions of vaccine efficacy and safety also influence vaccination willingness. Clear communication about the high efficacy rates of the HPV vaccine and its role in preventing HPV‐related cancers is crucial to building public confidence (Elenwo et al. 2023). The structure and timing of vaccination programs, including recommended ages for vaccination, impact uptake (Montalti et al. 2024). Flexible and accessible programs that align with individual schedules and life stages are important to improve vaccination rates. The implications of HPV vaccine hesitancy for public health are profound, underscoring the need for comprehensive strategies that address a range of influencing factors. Efforts to improve vaccine education, increase accessibility, and rebuild trust in the healthcare system are essential to counteract vaccine hesitancy. Clear, factual education campaigns should address misconceptions and promote the vaccine's benefits and safety. Engaging community leaders and tailoring messages to specific cultural contexts can also help build trust in the vaccine.

Future research directions should focus on understanding the complexities of HPV vaccine hesitancy across different demographic and socioeconomic groups. Longitudinal studies are needed to track changes in vaccine attitudes over time and to assess the impact of interventions on various populations. Specific areas for future research could include the effectiveness of tailored educational campaigns, the role of social media in spreading or countering misinformation, and the unique needs of underrepresented groups such as minorities and low‐income individuals. Research should also examine how socioeconomic factors, such as healthcare access and financial barriers, interact with vaccine hesitancy. Additionally, exploring community‐based and culturally sensitive programs could reveal intervention strategies that directly impact hesitancy. Finally, evaluating vaccine safety and efficacy on an ongoing basis will support public health messaging and reassure the public about vaccination benefits. This study has several limitations. Lack of public involvement in this conceptual analysis limits the study's real‐world relevance, as patient and public perspectives were not included. Future studies should incorporate patient and public input through focus groups or community consultations, especially in research addressing public health issues like vaccine hesitancy. Geographic limitations may also mean that findings may not be applicable to all regions. The use of self‐reported data may be subject to recall and social desirability biases, while cross‐sectional designs limit causal inference. Additionally, the absence of longitudinal data makes it difficult to assess trends over time.

## Conflicts of Interest

The authors declare no conflicts of interest.

### Peer Review

The peer review history for this article is available at https://www.webofscience.com/api/gateway/wos/peer‐review/10.1111/jan.16653.

## Data Availability

The data supporting this study's findings are openly available in the database mentioned in the study.
